# The Role of Trogocytosis in the Modulation of Immune Cell Functions

**DOI:** 10.3390/cells10051255

**Published:** 2021-05-19

**Authors:** Kensuke Miyake, Hajime Karasuyama

**Affiliations:** Inflammation, Infection & Immunity Laboratory, Advanced Research Institute, Tokyo Medical and Dental University (TMDU), Tokyo 113-8510, Japan; karasuyama.mbch@tmd.ac.jp

**Keywords:** trogocytosis, antibody-dependent cellular cytotoxicity (ADCC), T cell receptor (TCR), major histocompatibility complex (MHC), Fcγ receptor, NK receptor, cross-dressing, Th2 differentiation, intracellular bacteria

## Abstract

Trogocytosis is an active process, in which one cell extracts the cell fragment from another cell, leading to the transfer of cell surface molecules, together with membrane fragments. Recent reports have revealed that trogocytosis can modulate various biological responses, including adaptive and innate immune responses and homeostatic responses. Trogocytosis is evolutionally conserved from protozoan parasites to eukaryotic cells. In some cases, trogocytosis results in cell death, which is utilized as a mechanism for antibody-dependent cytotoxicity (ADCC). In other cases, trogocytosis-mediated intercellular protein transfer leads to both the acquisition of novel functions in recipient cells and the loss of cellular functions in donor cells. Trogocytosis in immune cells is typically mediated by receptor–ligand interactions, including TCR–MHC interactions and Fcγ receptor-antibody-bound molecule interactions. Additionally, trogocytosis mediates the transfer of MHC molecules to various immune and non-immune cells, which confers antigen-presenting activity on non-professional antigen-presenting cells. In this review, we summarize the recent advances in our understanding of the role of trogocytosis in immune modulation.

## 1. Introduction

**Figure 1:**
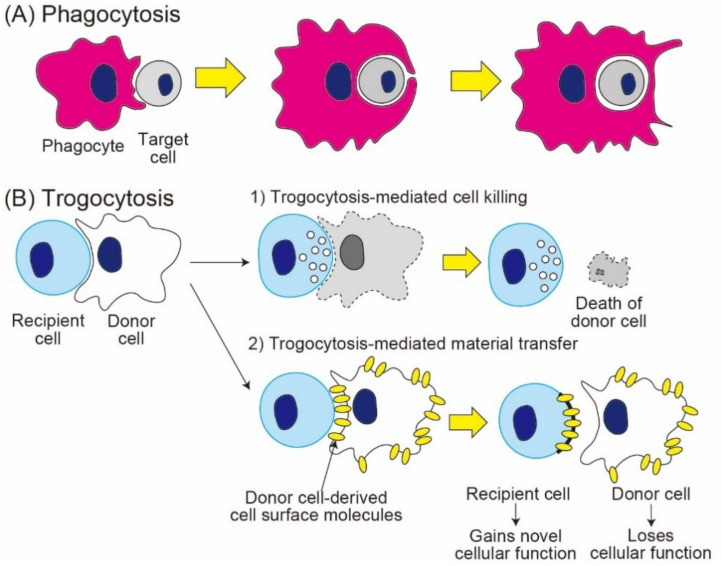
Characteristics of phagocytosis vs. trogocytosis. (**A**) In the process of phagocytosis, phagocytes swallow the whole cell body of target cells; (**B**) in the process of trogocytosis, recipient cells nibble the cell body of donor cells. Trogocytosis results in either (1) the death of target cells (trogocytosis-mediated cell death) or (2) the transfer of cell surface molecules, together with membrane patches, from donor cells to recipient cells (trogocytosis-mediated material transfer).

In the past decade, trogocytosis (named from the ancient Greek *trogo-*, which means gnaw or nibble) has been attracting increasing attention in the vast fields of biology research, including immunology, microbiology, neurology, and developmental biology [1,2,3,4,5,6,7]. Trogocytosis is an active process, in which one cell rapidly acquires the fraction of another cell in a cell contact-dependent manner [8] (Figure 1). Trogocytosis is typically distinguished from phagocytosis, which indicates engulfment of the entire cell body (Figure 1).

Trogocytosis was first documented in the field of microbiology in 1979. Brown discovered that *Naegleria fowleri*, known as “brain-eating amoeba,” nibbles mouse-embryo cells in a piecemeal fashion, a phenomenon termed trogocytosis [9]. In amoeba species, trogocytosis is a means of killing host cells to invade host tissues [10,11,12]. In the field of immunology, the transfer of major histocompatibility complex (MHC) proteins or immunoglobulins from B cells to T cells was reported in the 1970s [13,14]. In the early 1980s, host-derived MHC proteins were demonstrated to be acquired by donor-derived thymocytes in the system of bone marrow-chimeric mice [15,16]. In 2003, Joly and Hudrisier coined the term trogocytosis to explain the phenomenon of membrane protein transfer after immunological synapse formation in T cells, B cells, and natural killer (NK) cells [8]. To date, trogocytosis has been observed in various types of immune cells, including macrophages [17], dendritic cells (DCs) [18], neutrophils [19], basophils [20], and innate lymphoid cells (ILCs) [21]. Trogocytosis has also been reported in non-immune cells, including lymph node stromal cells [22] and mesenchymal stromal cells [23]. Furthermore, trogocytosis by microglia contributes to brain homeostasis by pruning axons to remodel neurons [24,25,26]. 

In the process of trogocytosis, recipient cells acquire membrane fractions, including cell surface molecules, from donor cells. Vanherbergen et al. reported that ~3% of cell surface receptors on donor cells are rapidly transferred to recipient cells in trogocytosis by NK cells [27]. Therefore, recipient cells can gain novel functions by acquiring molecules from donor cells, while donor cells may reduce their cellular functions by the deprivation of their cell surface molecules. Moreover, in some cases, trogocytosis even results in the death of donor cells via a process called trogoptosis [28]. Thus, trogocytosis can modulate the function of both donor and recipient cells [1,2,8,29,30,31,32]. In this review, we summarize the immune-modulatory effects of trogocytosis. First, we provide an overview of trogocytosis-mediated cell death. Second, we focus on trogocytosis triggered by ligand–receptor interactions, including T cell receptor (TCR)/MHC and Fcγ receptor/antibody-bound cell surface molecule interactions. Finally, we discuss the trogocytosis observed in other immune cells, including basophils, DCs, and innate lymphoid cells.

## 2. Trogocytosis-Mediated Cell Death

**Figure 2:**
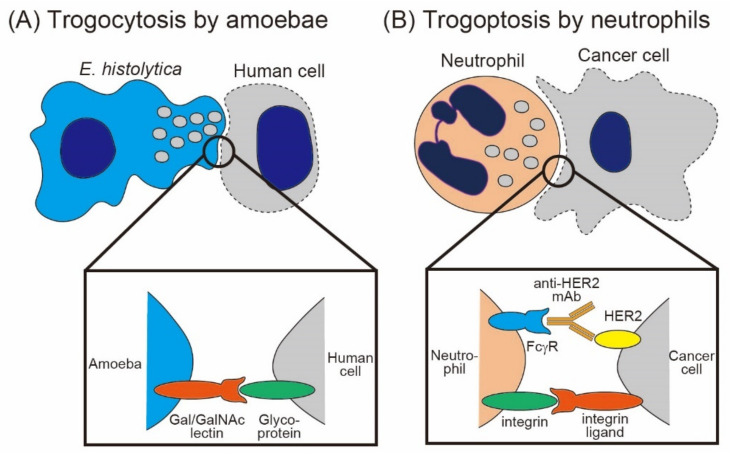
Trogocytosis-mediated cell death by amoebae and neutrophils. (**A**) Amoebae nibble human cells via trogocytosis, leading to the death of human cells. The interaction between amoebae and human cells is dependent on Gal/GalNAc lectin expressed on amoebae; (**B**) neutrophils nibble antibody-opsonized cancer cells via trogocytosis, resulting in cancer cell death (a process called trogoptosis). Trogoptosis by neutrophils is dependent on FcγR and Mac-1 integrin expressed on neutrophils.

As described above, trogocytosis by amoebae leads to the death of host cells [6]. Amoebic trogocytosis is mainly studied by using *Entamoeba histolytica*, a causative microbe of amoebiasis in humans. Ralston et al. reported that amoebae rapidly extract cell fragments from living Jurkat cells via trogocytosis, leading to the upregulation of the intracellular calcium concentration and the death of Jurkat cells [10] (Figure 2A). Importantly, amoebae perform trogocytosis-mediated cell killing only when target cells are alive, whereas amoebae perform phagocytosis of target cells when cells are dead [10,11]. Although similar processes are involved in both amoebic trogocytosis and phagocytosis, including Gal/GalNac lectin-mediated cell adhesion, requirement of actin, signaling through phsphoinositol-3 kinase (PI3K), and lysosomal acidification [10,12,33], several reports have implied that the molecular pathways in trogocytosis and phagocytosis are different [6,11]. A recent report demonstrated that AGC family kinase 1 (AGCK1), a molecule downstream of PI3K, is specifically involved in trogocytosis but not in phagocytosis [11], even though AGCK2 is involved in both. Furthermore, a cysteine protease inhibitor impairs amoebic trogocytosis but not phagocytosis, suggesting the possible involvement of a certain amoebic cysteine protease in trogocytosis [34]. Trogocytosis in amoebae would have been discussed in detail by other articles in this issue.

Mammalian immune cells can perform trogocytosis-mediated killing of pathogens. Mercer et al. reported that human neutrophils kill *Trichomonas vaginalis* parasites via trogocytosis [35]. When human neutrophils are co-cultured in vitro with *T. vaginalis* parasites, neutrophils rapidly nibble the cell body of *T. vaginalis*, leading to the death of parasites. As observed in trogocytosis by amoebae, human neutrophils trogocytose live parasites, but not heat-inactivated dead parasites. 

Moreover, trogocytosis-mediated cell death is utilized in antibody-dependent cellular cytotoxicity (ADCC) against cancer cells by neutrophils and macrophages [28,36,37] (Figure 2B). Matlung et al., identified that mouse and human neutrophils nibble antibody-opsonized cancer cells, leading to the necrotic cell death of cancer cells (a process designated as trogoptosis) [28]. Similar to amoebic trogocytosis, neutrophil trogocytosis and cytotoxicity against cancer cells are dependent on PI3K, a signaling molecule downstream of the Fcγ receptor. Trogoptosis by neutrophils is dependent on CD11b/CD18 (Mac-1) integrin-mediated conjugation between neutrophils and cancer cells [28,37]. Interestingly, “don’t eat-me signal” CD47 on cancer cells inhibits neutrophil trogoptosis via interaction with signal regulatory protein a (SIRPa), which provides mechanistic insight into the CD47-SIRPa checkpoint blockade [28,37]. Velumurugan et al., reported a similar trogoptosis phenomenon, in which mouse and human macrophages kill antibody-opsonized cancer cells [36]. Moreover, a recent report revealed that mouse and human vaginal neutrophils can eliminate sperms via trogocytosis, to prevent excess inflammation triggered by exogeneous sperms [38]. Taken together, the mechanisms of trogocytosis-mediated cell death can be operative in both amoeba parasites and mammalian immune cells, suggesting the evolutional conservation of this phenomenon in eukaryotic cells.

## 3. Trogocytosis Triggered by LIGAND–Receptor Interaction-1: TCR-Mediated Trogocytosis

### 3.1. Overview of Trogocytosis Triggered by TCR–MHC Interaction

In most cases, trogocytosis in immune cells requires ligand–receptor interactions [8]. TCR–MHC interaction is a well-characterized one that triggers trogocytosis. Upon stimulation of TCR on T cells by peptide–MHC complexes on antigen-presenting cells (APCs), an immunological synapse is formed between T cells and APCs. An immunological synapse consists of a central supramolecular activation cluster (cSMAC) enriched with TCR and intracellular signaling molecules, and a peripheral supramolecular activation cluster (pSMAC) enriched with integrins that surrounds cSMAC [39]. The formation of an immunological synapse results in the internalization of TCR and the transfer of peptide–MHC (pMHC) complexes, together with membrane fragments of APCs, onto the surface of T cells [40,41,42]. As observed in trogocytosis by amoebae, TCR-mediated trogocytosis is dependent on both actin polymerization and the TCR signaling pathway, including Src kinase and PI3K [43,44]. Moreover, the formation of an immunological synapse is considered to play a key role in TCR-mediated trogocytosis. Indeed, trogocytosis is abrogated by the blockade of either co-stimulatory molecules (CD28–CD80/CD86 interactions) or integrins (interactions between lymphocyte function-associated antigen 1 (LFA-1) and intercellular adhesion molecule-1 (ICAM-1)) [45]. Interestingly, both co-stimulatory molecules (e.g., CD80, CD86, and OX-40 ligand) and integrin ligands (e.g., ICAM-1) are also transferred, together with pMHC complexes [45,46,47]. These results imply that a wide variety of molecules present in the immunological synapse are transferred upon TCR-mediated trogocytosis. However, it is worth noting that only limited sets of cell surface molecules are selectively transferred in trogocytosis [48,49]. 

In general, CD4^+^ T cells capture peptide–MHC class II (pMHC-II) complexes from APCs, whereas CD8^+^ T cells acquire peptide–MHC class I (pMHC-I) complexes from APCs. Several reports indicate that CD4^+^ T cells can acquire both cognate pMHC-II and bystander pMHC-I complexes, possibly because of the localization of bystander pMHC-I in the proximity of cognate pMHC-II complexes [50,51,52]. Likewise, CD8^+^ T cells can acquire both cognate pMHC-I and bystander pMHC-II complexes [53].

Martínez-Martín et al. revealed that small GTPases, TC21 and RhoG, play central roles in TCR internalization and the trogocytosis of MHC-II upon immunological synapse formation between CD4^+^ T cells and APCs [44]. TC21 and RhoG also contribute to the trogocytosis of MHC-I in CD8^+^ T cells, indicating the essential roles of these small GTPases in TCR-mediated trogocytosis in both CD4^+^ and CD8^+^ T cells. Upon formation of an immunological synapse, TC21 and RhoG are internalized, together with TCR. Importantly, CD4^+^ T cells deficient of TC21 or RhoG fail to internalize TCR and capture MHC-II from APCs, demonstrating their role in TCR-mediated trogocytosis. An in vitro study using Jurkat cells further revealed that RhoG activation in T cells is dependent on TC21 and PI3K. Since TC21 is reported to activate the PI3K pathway [54], one may assume that the immunological synapse induces TC21 activation that, in turn, activates RhoG down-stream of PI3K, leading to the trogocytosis of pMHC complexes.

### 3.2. The Impact of TCR-Mediated Trogocytosis in CD4^+^ T Cell Functions

TCR–pMHC-II interaction results in TCR internalization and a subsequent display of pMHC-II on the surface of CD4^+^ T cells. Several reports have indicated that the trogocytosis of pMHC-II complexes prolongs the association between TCR and pMHC-II complexes, sustains TCR signaling, and promotes the survival of CD4^+^ T cells, even after the removal of APCs [55,56,57]. Furthermore, a recent report demonstrated that pMHC-II-dressed CD4^+^ T cells preferentially differentiate into GATA3^+^ Th2 cells that secrete IL-4 and IL-5, 72 h after the removal of APCs [57]. Given that weaker TCR signaling drives polarization toward Th2 cells [58], it can be assumed that weak and sustained TCR signaling triggered by acquired pMHC-II may favor Th2 differentiation. However, another report by Boccasavia et al. demonstrated that pMHC-II-dressed CD4^+^ T cells preferentially differentiate into regulatory T cells (Tregs) after presenting acquired antigens to other naïve T cells [59]. This apparent discrepancy may stem from the difference in the experimental systems they adopted. Further research is required to determine the fate of pMHC-II-dressed CD4^+^ T cells under more physiological conditions.

**Figure 3:**
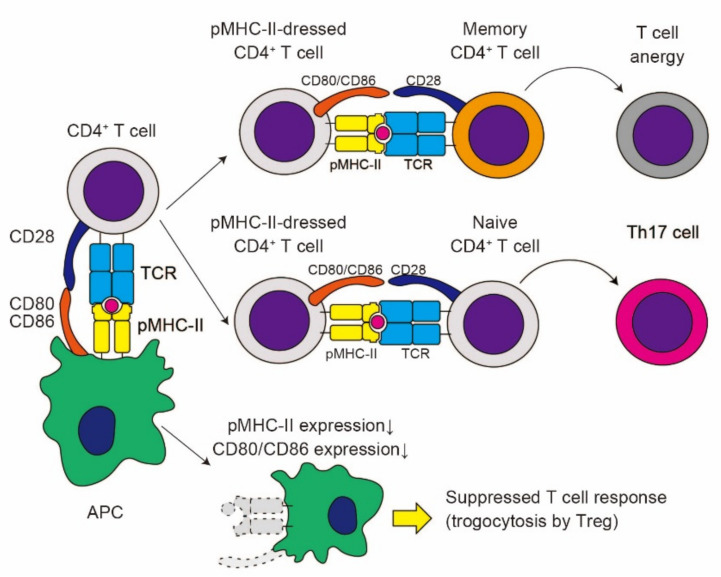
The impact of trogocytosis on CD4^+^ T cell responses. Upon formation of an immunological synapse, CD4^+^ T cells acquire pMHC-II complexes from APCs. pMHC-II-dressed CD4^+^ T cells can present antigens to memory CD4^+^ T cells, resulting in T cell anergy, while their antigen presentation to naïve CD4^+^ T cells may result in differentiation into pathogenic Th17 cells. Moreover, trogocytosis by Tregs can reduce the expression of pMHC-II and co-stimulatory molecules on APCs, leading to the suppression of the CD4^+^ T cell responses elicited by interaction with APCs.

Several reports have indicated that CD4^+^ T cells with acquired pMHC-II complexes can further present antigens to cognate CD4^+^ T cells, leading to CD4^+^ T cell–T cell interactions [46,59,60,61,62,63]. When MHC-II-dressed CD4^+^ T cells present antigens to one another, antigen presentation leads to the negative regulation of CD4^+^ T cell responses [32] (Figure 3). Indeed, Tsang et al., revealed that mouse CD4^+^ T cells that acquire pMHC-II complexes from APCs induce either the apoptosis or anergy of CD4^+^ T cells, possibly through mutual antigen presentation among pMHC-II-dressed CD4^+^ T cells [60]. Helft et al., reported that pMHC-II-dressed CD4^+^ T cells present antigens to memory CD4^+^ T cells, which suppresses the proliferation of memory CD4^+^ T cells [62]. Thus, antigen presentation by pMHC-II-dressed CD4^+^ T cells form a negative feedback loop that may limit excess T cell responses. On the contrary, Boccasavia et al., recently demonstrated that pMHC-II-dressed CD4^+^ T cells present antigens to naïve CD4^+^ T cells, which results in differentiation into pathogenic Th17 cells from naïve CD4^+^ T cells [59] (Figure 3). In a model of experimental autoimmune encephalitis (EAE), mice deficient of RhoG, an essential molecule for TCR-mediated trogocytosis, display ameliorated Th17 inflammation, which results in resistance to EAE development. Therefore, antigen presentation by pMHC-II-dressed CD4^+^ T cells may favor pro-inflammatory Th17 polarization under certain circumstances.

In addition to the impact on CD4^+^ T cells, TCR-mediated trogocytosis regulates the function of APCs. A recent report by Akkaya et al., revealed that induced regulatory T cells (iTregs) form intense interactions with DCs, leading to a greater capacity for trogocytosis compared to activated CD4^+^ T cells [64]. Thus, iTregs reduce the expression of cognate pMHC-II complexes on the surface of DCs, resulting in the diminished antigen-presenting capacity of DCs (Figure 3). Moreover, iTregs downregulate the expression of CD80 and CD86 on DCs by means of stripping of these co-stimulatory molecules during trogocytosis [65]. Thymus-derived regulatory T cells (tTregs) capture CD80 and CD86 from DCs via trans-endocytosis mediated by cytotoxic T lymphocyte-associated antigen-4 (CTLA-4), leading to impaired co-stimulation by DCs [66,67]. In addition, CTLA-4 on Tregs reportedly extract co-stimulatory molecules from DCs via trogocytosis [68], suggesting the possible role of trogocytosis in CTLA-4-mediated suppression. Additionally, tTregs reduce the expression of CD70 on DCs via trogocytosis mediated by the interaction with CD27 on Tregs, which results in the inhibition of Th1 priming by DCs [69]. Tregs also downregulate CD137 ligand on DCs via CD137-mediated trogocytosis, leading to the suppression of T cell activation by DCs [70]. Collectively, trogocytosis in Tregs suppresses DC functions via multiple pathways.

### 3.3. The Impact of TCR-Mediated Trogocytosis on CD8^+^ T Cell Functions

**Figure 4:**
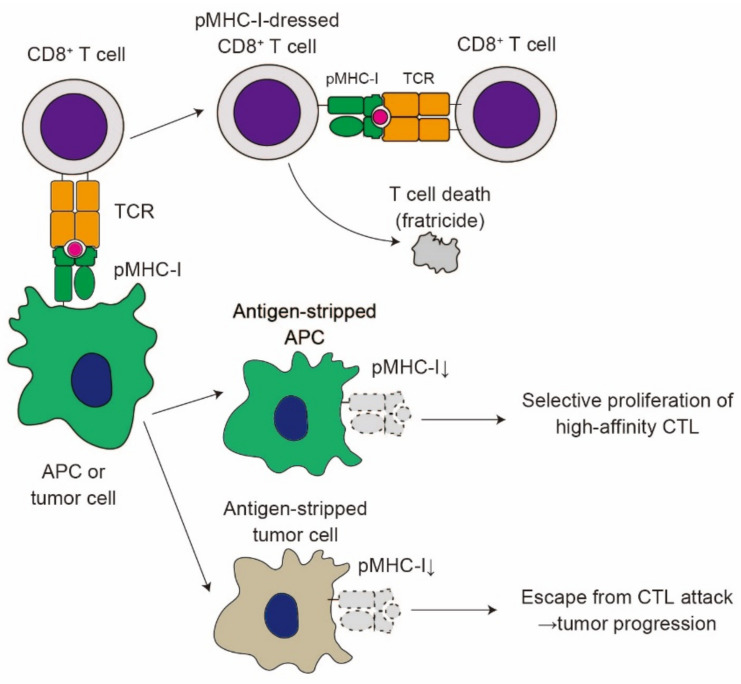
The impact of trogocytosis on CD8^+^ T cell responses. Upon formation of immunological synapse, CD8^+^ T cells acquire pMHC-I complexes from APCs or tumor cells. pMHC-I-dressed CD8^+^ T cells are susceptible to attack from other CD8^+^ T cells, resulting in cell lysis (fratricide). Trogocytosis reduces the expression of pMHC-I on APCs (antigen stripping). Antigen stripping in APCs favors the selective proliferation of high-affinity CTL. On the contrary, antigen stripping in tumor cells contributes to the escape from CTL attack, leading to tumor progression.

CD8^+^ T cells with pMHC-I complexes acquired from APCs via trogocytosis suppress T cell responses through multiple pathways. Huang et al. reported that pMHC-I-dressed CD8^+^ T cells are sensitive to the cell lysis by other CD8^+^ T cells, a phenomenon called CD8^+^ T cell fratricide [40] (Figure 4). A later report demonstrated that CD8^+^ T cell fratricide can be observed at an extremely high antigen concentration [71], suggesting that the fratricide phenomenon occurs only in the presence of a high antigen concentration. A similar mechanism of fratricide was recently reported in the trogocytosis of T cells bearing chimeric antigen receptors (CARs) [72], which is further discussed below. CD8^+^ T cells also capture CD80 from APCs through trogocytosis [73]. The acquisition of CD80 by memory CD8^+^ T cells plays a rather suppressive role in their expansion and IL-2 production, suggesting the regulatory role of trogocytosis in recall immune responses. 

Trogocytosis by CD8^+^ T cells takes place either when APCs prime CD8^+^ T cells or when CD8^+^ T cells attack target cells, including tumor cells [74,75]. In their interaction with APCs, CD8^+^ T cells strip pMHC-I complexes from these APCs, which may favor the selective proliferation of CD8^+^ T cells with high-affinity TCR, leading to affinity maturation of CD8^+^ T cells [76] (Figure 4). Likewise, CD8^+^ T cells strip pMHC-I complexes from tumor cells [77]. Chung et al. demonstrated that cytotoxic lymphocytes (CTLs) with high-avidity TCR efficiently lyse melanoma cells, while low-avidity CTLs strip pMHC-I complexes from melanoma cells via trogocytosis, leading to the escape from target cell lysis by high-avidity CTLs (Figure 4). Thus, the trogocytosis of pMHC-I complexes from tumor cells blocks CD8^+^ T cell responses against tumors. Several reports have demonstrated that CD86 and HLA-G expression on T cells indicates the poor prognosis of multiple myeloma [78,79,80]. Given that CD86 and HLA-G are transferred from tumor cells via trogocytosis [79], trogocytosis of these molecules by CD8^+^ T cells may play a suppressive role in tumor immune responses.

Trogocytosis-mediated antigen stripping has recently been reported in CAR T cells [72]. CAR T cells are engineered T cells that express high-affinity variable fragments of antibody (single-chain variable fragment (scFv)) fused with an intracellular domain for signal transduction [81,82]. Therapies using patient-derived CAR T cells display remarkable efficacy against chemotherapy-resistant or refractory B cell malignancies. However, relapse can be observed in a large proportion of patients, and many patients relapse with antigen-low tumors, leading to escape from CAR T cell therapy [81,82]. Hamieh et al., revealed, using a mouse model of leukemia, that the insufficient infusion of CAR T cells causes the trogocytosis of target antigens from tumor cells to CAR T cells [72]. Trogocytosis by CAR T cells reduces the antigen density on target tumor cells, leading to the reduced efficacy of CAR T cell therapy. Moreover, trogocytosis-experienced CAR T cells display an exhausted phenotype and are susceptible to being killed by other T cells (fratricide). These results indicate that tumor cells exploit trogocytosis for escape from CAR T cell therapy.

## 4. Trogocytosis Triggered by Ligand–Receptor Interaction-2: FcγR-Mediated Trogocytosis 

**Figure 5:**
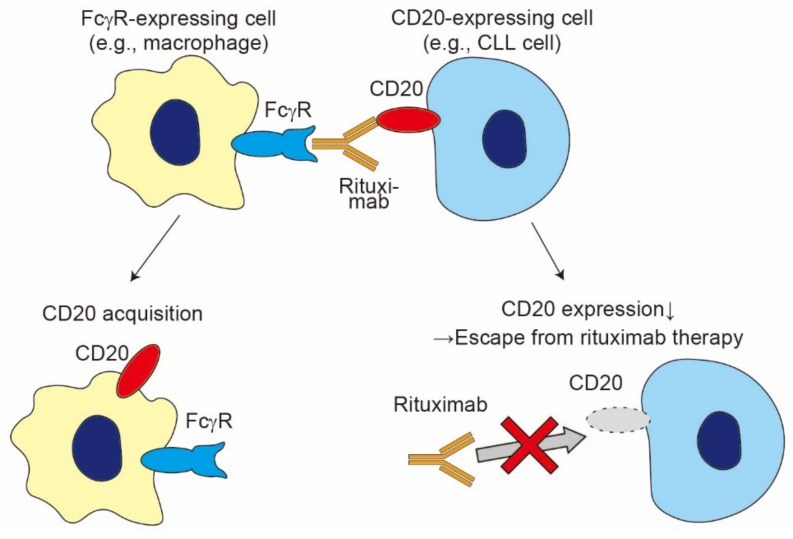
FcγR-mediated trogocytosis. In rituximab therapy, FcγR-expressing cells (e.g., macrophages) capture antibody-opsonized cancer cells (e.g., CLL cells), which leads to either the acquisition of CD20 by FcγR-expressing cells or a reduction in the expression of CD20 in cancer cells and escape from rituximab therapies.

Immunotherapeutic monoclonal antibodies (mAbs) are utilized to treat a wide variety of disorders, including cancer and autoimmune diseases [83]. After the administration of mAbs, Fcγ receptor (FcγR)-expressing cells, including monocytes, macrophages, neutrophils, and NK cells, can extract mAb-bound cell surface molecules from target cells via trogocytosis, leading to the reduced efficacy of mAb-based therapies [3,84] (Figure 5). As observed in TCR-mediated trogocytosis, FcγR-mediated trogocytosis occurs rapidly (within 1 h), accompanies the transfer of membrane patches, and requires actin polymerization [17,85,86,87,88]. 

Several reports have suggested that bystander cell surface molecules are also transferred in FcγR-mediated trogocytosis [89,90,91]. Rossi et al., revealed that the infusion of epratuzumab (anti-CD22 mAb) reduces not only the expression of CD22, but also the expression of other cell surface molecules, including CD19, CD21, and CD79b, on B cells [89], which may favor the efficient inhibition of pathogenic B cell functions in the treatment of systemic lupus erythematosus (SLE).

It remains controversial whether FcgR-mediated trogocytosis requires signaling through Src kinase and PI3K, since conflicting results have been obtained depending on the experimental conditions adopted [17,85]. Although several reports have indicated that FcγR-mediated trogocytosis is rarely observed by incubation on ice [85,86], a recent report suggested that FcγR-mediated trogocytosis is also operative at 4 °C [91], suggesting the presence of multiple mechanisms for FcγR-mediated trogocytosis.

To date, an array of therapeutic mAbs have been reported to cause trogocytosis, including rituximab, epratuzumab, daclizumab, cetuximab, trastuzumab, and daratumumab [86,89,92,93,94,95,96]. Among these mAbs, anti-CD20 mAbs, including rituximab (RTX), ofatumumab (OFA), and obinutuzumab (OBZ), are the best-characterized ones to cause trogocytosis-mediated antigen stripping from tumor cells [85,86,92,93]. RTX infusion in patients with chronic lymphocytic leukemia (CLL) rapidly decreases the surface expression of CD20 on tumor cells via trogocytosis, resulting in the incomplete lysis of tumor cells [85,97]. Interestingly, regular-dose anti-CD20 mAb therapy promotes the excess consumption of complement system during the first antibody infusion [97]. Such complement depletion, along with a reduction of surface CD20 expression, induces resistance to anti-CD20 mAb therapy [97,98]. Taylor et al., proposed that low-dose anti-CD20 mAb therapy can prevent both excess complement consumption and trogocytosis-mediated CD20 reduction, leading to the increased efficacy in tumor cell death [3]. Indeed, Williams et al., demonstrated that low-dose RTX treatment reduces the occurrence of trogocytosis and enhances the clearance of circulating CLL cells [99]. 

Beum et al., reported that FcγRI on monocytes is internalized after FcγR-mediated trogocytosis [92]. Interestingly, a recent report by Pinney et al., revealed that macrophages show a reduced capacity for phagocytosis (termed hypophagia) after rituximab treatment, possibly through the reduction of surface FcγRI in macrophages [100]. Although it remains to be elucidated whether trogocytosis is associated with hypophagia, it can be assumed that the trogocytosis-mediated loss of FcγRI results in the hypophagia of macrophages, which provides another possible mechanism for resistance to RTX therapies. 

Taken together, FcγR-mediated trogocytosis contributes to the resistance to mAb-based therapies, mainly through the loss of target antigens. However, it should be noted that FcγR-mediated trogocytosis in neutrophils or macrophages can enhance target cell death via trogoptosis [28,36], as described in Section 2. On the contrary, Valgardsdottir et al., revealed that RTX-mediated trogocytosis has little effect on tumor cell death [93]. Further research is required to elucidate which factor(s) causes the different consequences of FcγR-mediated trogocytosis.

## 5. Trogocytosis Triggered by Ligand–Receptor Interaction-3: NK Receptor-Mediated Trogocytosis 

In 2001, three reports demonstrated that the formation of an immunological synapse between NK cells and target cells results in the trogocytosis of pMHC-I complexes and membrane fragments from target cells to NK cells [101,102,103]. MHC-I acquisition by NK cells is mediated by inhibitory NK cell receptors that recognize MHC-I, including Ly49 receptor (mouse) and killer Ig-like receptor (KIR)2DL1 (human). As observed in TCR-mediated trogocytosis, the trogocytosis of pMHC-I complexes accompanies the transfer of membrane patches, which is dependent on Src kinase-signaling and actin polymerization [104]. Furthermore, a subsequent report revealed that the transfer is bidirectional, in that inhibitory NK cell receptors are also transferred from NK cells to target cells [27]. 

In mouse studies, it has been revealed that Ly49A^+^ NK cells that acquire external MHC-I (H-2D^d^) display reduced cytotoxic functions, possibly due to *cis* interactions between inhibitory Ly49A and MHC-I on NK cells [102,103]. Similarly, human activated NK cells capture HLA-G from tumor cells, and NK cells that acquire HLA-G cease proliferation and reduce their cytotoxic capacity [105]. Moreover, HLA-G-dressed NK cells suppress the cytotoxic function of other NK cells expressing ILT2, the inhibitory receptor for HLA-G, which possibly leads to the immune escape of HLA-G-expressing tumor cells. These observations suggest that inhibitory NK receptor-mediated trogocytosis suppresses NK cell responses.

Moreover, later reports have demonstrated that NK cells acquire ligands for activating NK cell receptors via trogocytosis [106,107]. Nakamura et al. revealed that the natural killer group 2 membrane D (NKG2D) on NK cells extracts Rae-1, a ligand for NKG2D, from target cells [106]. Interestingly, Rae-1-dressed NK cells are further lysed by other NK cells expressing NKG2D (NK cell fratricide), which may act as a negative regulator of activated NK cells. Miner et al. reported that another activating NK cell receptor Ly49H also captures a ligand for Ly49H, m157 [107]. The acquisition of m157 on NK cells rather blocks NK cell effector functions mediated by Ly49H, possible through cis interactions between m157 and Ly49H on NK cells. Taken together, activating NK cell receptor-mediated trogocytosis can play regulatory roles in NK cell functions.

## 6. Trogocytosis-Mediated MHC Transfer

### 6.1. Overview of the Trogocytosis-Mediated Transfer of MHC Molecules

As described in Section 3, T cells extract MHC molecules from APCs via TCR-mediated trogocytosis. In addition, several reports have demonstrated that various cell types other than T cells acquire either pMHC-I or pMHC-II molecules via trogocytosis. Professional APCs such as DCs can capture the pMHC-I complexes generated by and expressed on other APCs via trogocytosis, enabling them to present antigens to CD8^+^ T cells without synthesizing peptides by themselves (a process called cross-dressing) [108,109,110]. On the contrary, non-professional APCs, including NK cells, basophils, and lymph node stromal cells, can capture the pMHC-II complexes expressed on APCs via trogocytosis, enabling them to present antigens to CD4^+^ T cells [20,22,111]. However, it remains to be elucidated what triggers the trogocytosis of MHC molecules. Providing that trogocytosis is generally triggered by receptor–ligand interactions, it can be assumed that some receptors for MHC molecules may trigger trogocytosis in these cells. Further research is required to elucidate the molecular mechanisms underlying trogocytosis-mediated MHC transfer. In this section, we summarize the roles of pMHC-I cross-dressing by DCs and pMHC-II acquisition by non-professional APCs.

### 6.2. Transfer of pMHC-I Complexes between DCs (Cross-Dressing)

DCs present antigens to CD8^+^ T cells via two major pathways [112,113]: One is a direct pathway for endogenous antigens, which are generated typically when DCs are virally infected; the other is a cross-presenting pathway for exogeneous antigens, including those released from dying cells, in which the cytosolic peptides derived from exogeneous antigens are transported to the endoplasmic reticulum (ER) via transporters associated with antigen processing (TAP). 

Trogocytosis can confer the third antigen presentation pathway on DCs, which is called the cross-dressing pathway. In this pathway, DCs capture pMHC-I complexes from target cells and present borrowed pMHC-I to CD8^+^ T cells. Dolan et al. demonstrated that DCs acquire OVA peptide-loaded MHC-I from dying cells, leading to the activation of OVA peptide-specific CD8^+^ T cells [108]. Wakim and Beaven revealed that cross-dressed DCs play key roles in CD8^+^ T cell responses in viral infection models [109]. Recent reports have also indicated the importance of cross-dressed DCs in allograft rejection models [110,114]. These data collectively suggest that DCs utilize trogocytosis for the induction of efficient CD8^+^ T cell responses. Cross-dressing and is impact on T cell responses would have been discussed by other articles in this issue.

### 6.3. pMHC-II Transfer from APCs to Immune Cells Other Than T Cells

**Figure 6:**
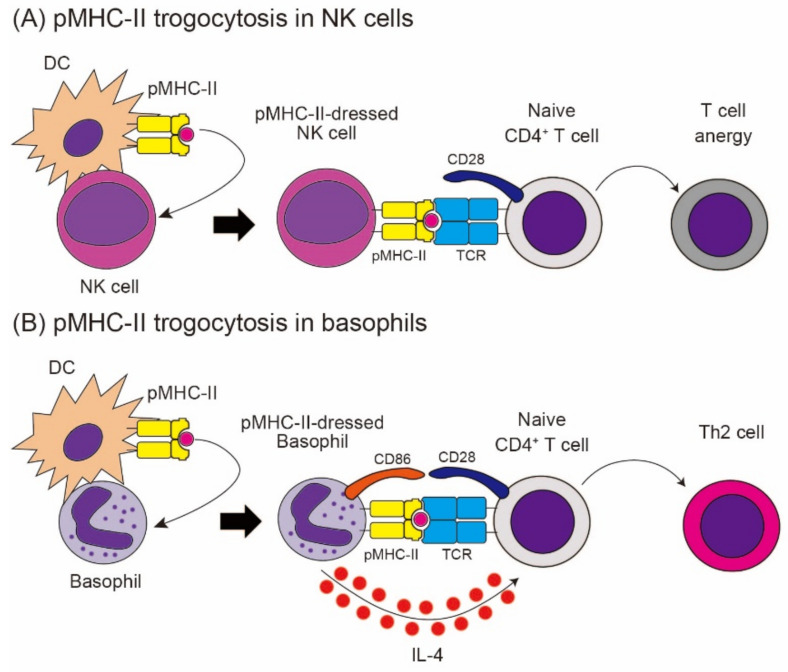
The trogocytosis of pMHC-II in NK cells and basophils. (**A**) NK cells acquire pMHC-II from APCs via trogocytosis. pMHC-II-dressed NK cells rather suppress CD4^+^ T cell responses, possible because of their low expression of co-stimulatory molecules. (**B**) Basophils acquire pMHC-II from APCs via trogocytosis. pMHC-II-dressed basophils present antigens to naïve CD4^+^ T cells, leading to T cell proliferation and differentiation into Th2 cells with the aid of the IL-4 produced by basophils.

Growing evidence suggests that non-professional APCs play important roles in modulating immune responses [115,116,117]. The trogocytosis of pMHC-II complexes from APCs can confer an antigen-presenting capacity on non-APCs, including NK cells, basophils, and lymph node stromal cells. In these cells, pMHC-II complexes are selectively transferred from DCs, while other cell surface molecules, including co-stimulatory molecules, are rarely transferred, unlike TCR-dependent trogocytosis [20,22,111]. Therefore, the consequence of MHC-II acquisition via trogocytosis in terms of T cell functions depends on the endogenous expression of co-stimulatory molecules on MHC-II-dressed non-APCs. Nakayama et al. reported that NK cells acquire pMHC-II complexes from DCs and present antigens to T cells [111] (Figure 6). Antigen presentation by MHC-II-dressed NK cells rather suppresses the proliferation of CD4^+^ T cell, possibly because of their little expression of co-stimulatory molecules on NK cell surface. Dubrot et al. reported that afferent lymphatic epithelial cells (LECs) capture pMHC-II complexes from DCs, and antigen presentation by MHC-II-dressed LECs induces T cell apoptosis and suppresses the proliferation of activated CD4^+^ T cells, since LECs lack the endogenous expression of co-stimulatory molecules [22]. By contrast, Miyake et al. identified that basophils acquire pMHC-II complexes from DCs, and present acquired antigens to naïve T cells, leading to the proliferation of CD4^+^ T cells and the differentiation into Th2 cells [20] (Figure 6). Basophils endogenously express co-stimulatory molecule CD86, which enables MHC-II-dressed basophils to enhance CD4^+^ T cell proliferation, unlike NK cells and LECs. 

Basophils are the least common granulocytes, which represent less than 1% of peripheral blood leukocytes. The functional significance of basophils has long been an enigma, partly because of their rarity and the paucity of analytical tools for basophil research. However, recent research has revealed that basophils play significant roles in various immune responses, including chronic allergic inflammation and protective immunity against parasites [118,119,120,121]. Moreover, basophils influence acquired immunity by triggering Th2 differentiation. Of note, mouse and human basophils rapidly secrete large amounts of IL-4 in response to various stimuli [122]. Therefore, it has been assumed that basophils act as accessory cells for Th2 differentiation, in which APCs present antigens to naïve T cells, while basophils provide IL-4 to promote their differentiation into Th2 cells. Supporting this notion, in a papain-induced skin inflammation model, basophils are transiently recruited to draining lymph nodes, and the depletion of basophils impairs Th2 cell differentiation there [123]. Furthermore, subsequent reports have demonstrated that basophils express MHC-II on their cell surface and present antigens to naïve T cells, leading to differentiation into Th2 cells, in concert with IL-4 secreted from basophils [124,125,126]. They also revealed that depletion of basophils but not DCs impair Th2 differentiation [124,125]. However, later reports have suggested that basophils have little potential for antigen presentation, since they have little expression of antigen-processing machinery, including cathepsin S and invariant chain [127,128]. Moreover, several reports have argued that DCs, rather than basophils, play important roles in Th2 differentiation [127,129]. Trogocytosis-mediated pMHC-II transfer appears to partly reconcile the discrepancy observed in the above-mentioned reports [20]. Basophils can acquire pMHC-II complexes from DCs and can present antigens to T cells, without antigen processing. Trogocytosis-mediated basophil–DC interplay confers antigen-presenting activity on basophils, which facilitates Th2 differentiation. Therefore, it can be assumed that the extent of basophil–DC interaction may determine the relative contribution of basophils to Th2 differentiation.

Dudeck et al. demonstrated that mast cells can also acquire MHC-II from DCs and can prime T cell responses [130]. Furthermore, mast cell–DC synapses enhance antigen transfer from mast cells to DCs [131]. Therefore, trogocytosis-mediated mast cell–DC interplay may facilitate cutaneous T cell responses [132]. Oliphant et al. revealed that group 2 innate lymphoid cells (ILC2s) also express MHC-II on their cell surface, partly via trogocytosis [21]. Interaction between MHC-II-expressing ILC2s and CD4^+^ T cells activates T cells to produce IL-2, which, in turn, activates ILC2s to produce IL-5 and IL-13. Taken together, trogocytosis-mediated MHC-II transfer from APCs to other types of cells confers antigen-presenting capacity in non-APCs, leading to the modulation of CD4^+^ T cell immune responses.

## 7. Immune Cell Trogocytosis Exploited by Pathogen Microbes

Recent reports have revealed that the trogocytosis phenomenon is utilized by intracellular bacteria for their spread in hosts [133,134]. Steele et al., demonstrated that macrophages infected with *Francisella tularensis* bacteria transfer a cytosolic compartment containing viable bacteria to other uninfected macrophages, leading to the spread of intracellular bacteria. Interestingly, *F. tularensis* infection in macrophages increases the trogocytosis-mediated transfer of cell membrane and cell surface molecules (e.g., MHC), suggesting that intercellular bacterial transfer is associated with trogocytosis [133]. Indeed, uninfected macrophages extract cell fragments, including viable *F. tularensis*, from infected macrophages in a cell contact-dependent manner [133,134], suggesting the involvement of trogocytosis in bacterial spreading. After the transfer of bacteria, *F. tularensis* can be detected in double-membraned vesicles, which is composed of a recipient cell-derived membrane (outer layer) and a donor cell-derived membrane (inner layer) [134]. *F. tularensis* bacteria escape from double-membraned vesicles by using a Type 6 secretion system, and they spread to the cytosol of recipient cells [134]. Collectively, trogocytosis in macrophages is exploited by *F. tularensis* pathogens for their spreading. Of note, it has been demonstrated that several types of intracellular pathogens are transferred directly between immune cells [135,136,137]. Therefore, it can be postulated that a wide variety of intracellular pathogens utilize trogocytosis by immune cells for their expansion in host cells.

During the infection of avian H5N1 influenza virus, B cells acquire α2,3 sialic acid, the receptor for avian flu, from monocytes via trogocytosis, thus facilitating the spread of influenza virus [138,139]. Interestingly, the trogocytosis-mediated transfer of α2,3 sialic acid is increased by H5N1 influenza infection, suggesting that trogocytosis is also exploited by avian influenza virus. Similarly, NK cells acquire CD21, the receptor for Epstein–Barr virus (EBV), from target cells [140], which may also facilitate EBV spreading. The regulation of trogocytosis could be one measure for preventing the spread of pathogens, including intracellular bacteria and H5N1 influenza virus.

## 8. Conclusions

Immune cells communicate with one another to maintain homeostasis and to eliminate pathogen invasion. Communication among immune cells typically relies on receptor–ligand interactions, including TCR–MHC interactions. To efficiently exchange information, T cells and APCs form a specialized structure called an immunological synapse. It is becoming increasingly recognized that the formation of an immunological synapse triggers intercellular protein transfer from APCs to T cells via the process of trogocytosis. Trogocytosis-mediated protein transfer can be observed in other receptor–ligand interactions, including FcγR- and NK cell receptor-mediated trogocytosis. Trogocytosis can result in both a gain-of-function effect on recipient cells and a loss-of-function effect on donor cells, thus modulating a wide variety of immune reactions. The trogocytosis-mediated transfer of MHC molecules has been observed in various immune and non-immune cells, and confers antigen-presenting activity in non-APCs, leading to the modulation of T cell immune responses. Furthermore, trogocytosis by immune cells is exploited by pathogens. Therefore, the regulation of trogocytosis could be a good target for treating a wide variety of diseases, including cancer, autoimmune diseases, allergic diseases, and various infectious diseases.
